# Case Report: Dacomitinib is effective in lung adenocarcinoma with rare EGFR mutation L747P and brain metastases

**DOI:** 10.3389/fonc.2022.863771

**Published:** 2022-08-09

**Authors:** Yibin Li, Weixi Guo, Bin Jiang, Chengkun Han, Feng Ye, Jingxun Wu

**Affiliations:** ^1^ Department of Medical Oncology, The First Affiliated Hospital of Xiamen University, Xiamen, China; ^2^ Department of Thoracic Surgery, The First Affiliated Hospital of Xiamen University, Xiamen, China; ^3^ Department of Neurology, The First Affiliated Hospital of Xiamen University, Xiamen, China; ^4^ Department of Radiology, The First Affiliated Hospital of Xiamen University, Xiamen, China

**Keywords:** Dacomitinib (PubMed CID: 11511120), lung cancer, brain metastasis, EGFR, L747P mutation

## Abstract

Due to the low incidence of rare EGFR mutation, its response to EGFR-TKI has not been fully investigated. L747P is a rare EGFR mutation in EGFR exon 19. Previous case reports showed that patients with EGFR L747P mutation responded to afatinib treatment. However, we encountered a patient with EGFR L747P who was resistant to afatinib but responded to dacomitinib. It is the first case report of the effective application of dacomitinib in a patient with L747P mutation and BMS, and the efficacy of BMS achieved PR.

## Introduction

Predictive biomarkers in advanced non-small-cell lung cancer (NSCLC) include sensitizing epidermal growth factor receptor (EGFR) mutations, ALK rearrangements, ROS1 rearrangements, BRAF V600E point mutations, METex14 skipping mutations, NTRK1/2/3 gene fusions, and RET rearrangements (1). The presence of EGFR exon 19 deletion or exon 21 L858R mutation suggests a potential benefit from EGFR tyrosine kinase inhibitor (EGFR-TKI) therapy; thus, these mutations are referred to as sensitizing EGFR mutations. About 47.6% of NSCLC in Chinese populations harbor somatic mutations in the tyrosine kinase domain of the EGFR gene, mostly consisting of in-frame deletions of exon 19 (36.7%) and L858R substitutions in exon 21 (33.4%) (2). However, because of the low incidence of rare EGFR mutation, its response to EGFR-TKI has not been fully investigated. L747P is a rare EGFR mutation in EGFR exon 19. In previous case reports, patients with the EGFR L747P mutation responded to afatinib treatment (3, 4). Incidentally, we encountered a patient with EGFR L747P who was resistant to afatinib. The patient ultimately responded well to dacomitinib and had a significant clinical benefit on developed BMS.

## Case presentation

**Figure 1:**
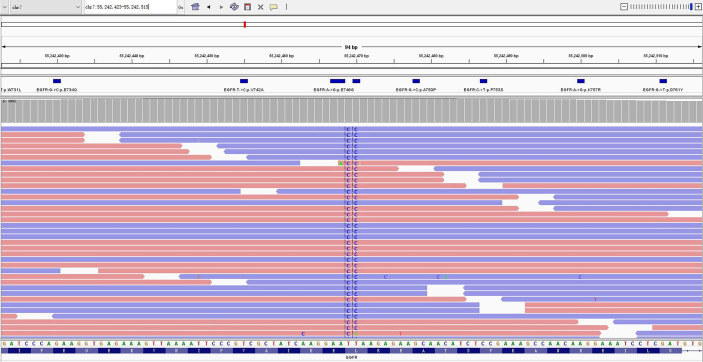
NGS panel results showed EGFR mutation L747P (2239-2240 TT>CC) in exon 19.

In April 2020, a 66-year-old Chinese woman with no smoking history was found to have a pulmonary mass in the right lower lobe of her lung by chest computed tomography (CT) scan during physical examination. The patient was subsequently hospitalized in the Department of Thoracic Surgery. Enhanced CT scan of the chest revealed a nodular lesion on the lower lobe of the right lung, the boundary is unclear, and the range of the central plane is about 5.0 cm × 4.2 cm; enhanced CT of the abdomen, emission CT bone scan, and brain magnetic resonance imaging (MRI) showed that no distant metastasis was found. After eliminating surgical contraindications by perfecting preoperative examinations, on 12 May 2020, the patient underwent thoracoscopic right lower lobectomy and systemic lymph node dissection. Postoperative pathology prompted invasive adenocarcinoma and postoperative staging is stage pT2bN2M0 IIIA. Next-generation sequencing (NGS) was performed with panels covering nine lung cancer-related genes (namely, ALK, BRAF, EGFR, ERBB2, KRAS, MET, NTRK, RET, and ROS1), and PD-L1 (Ventana SP263) expression detection in tumor tissue paraffin section (30% neoplastic cell) found EGFR exon 19 L747P mutation (EGFR NM_005228.3 Exon19 c.2239_2240del TTinsCC p.L747P) (**Figure 1**) with a mutation abundance of 14.21% and PD-L1 expression was greater than 10% in tumor cells (TCs). After the operation, the patient did not receive any antitumor therapy due to personal refusal.

**Figure 2:**
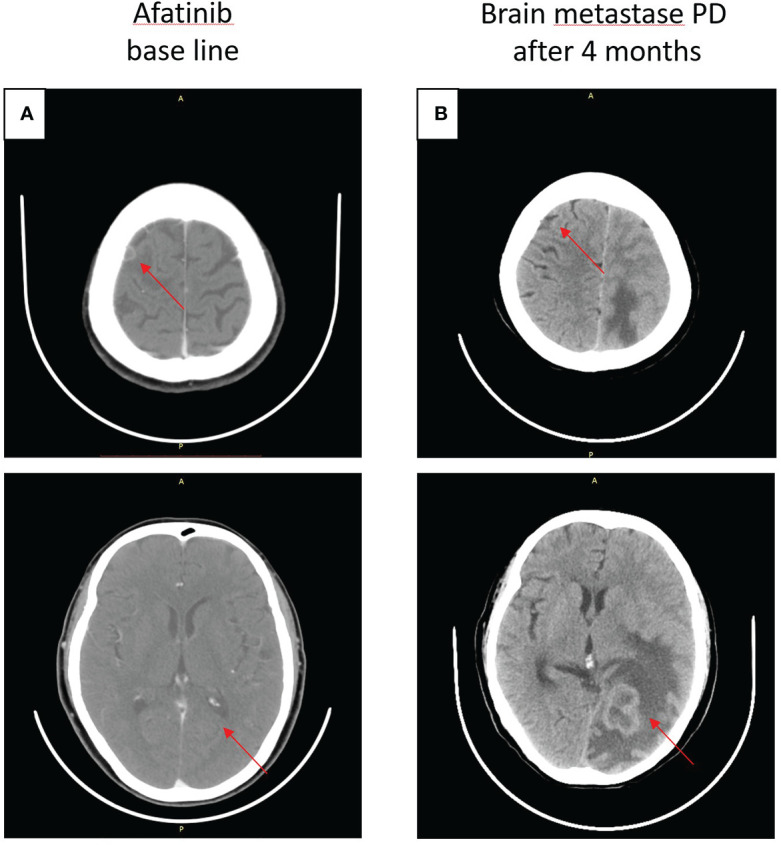
Craniocerebral CT about clinical response to afatinib therapy at different times. **(A)** Baseline before afatinib treatment. **(B)** Brain metastases progressive disease (PD) after 4 months.

On 14 July 2020, a CT scan was taken after the seizure of the patient, and the result revealed brain metastases (BMS) in the right frontal lobe of the brain, and then she accepted brain radiotherapy, which started from 20 July 2020, with a dose of 500 cGy/f, once a day, five times a week, and a planned target volume tumor absorbed dose (PTV DT) was 5000 cGy/10f. Subsequently, the patient started oral afatinib 40 mg daily as first-line therapy on 4 August 2020 (**Figure 2A**), and achieved stable disease for 7 months until January 2021 (**Figure 2B**).

**Figure 3:**
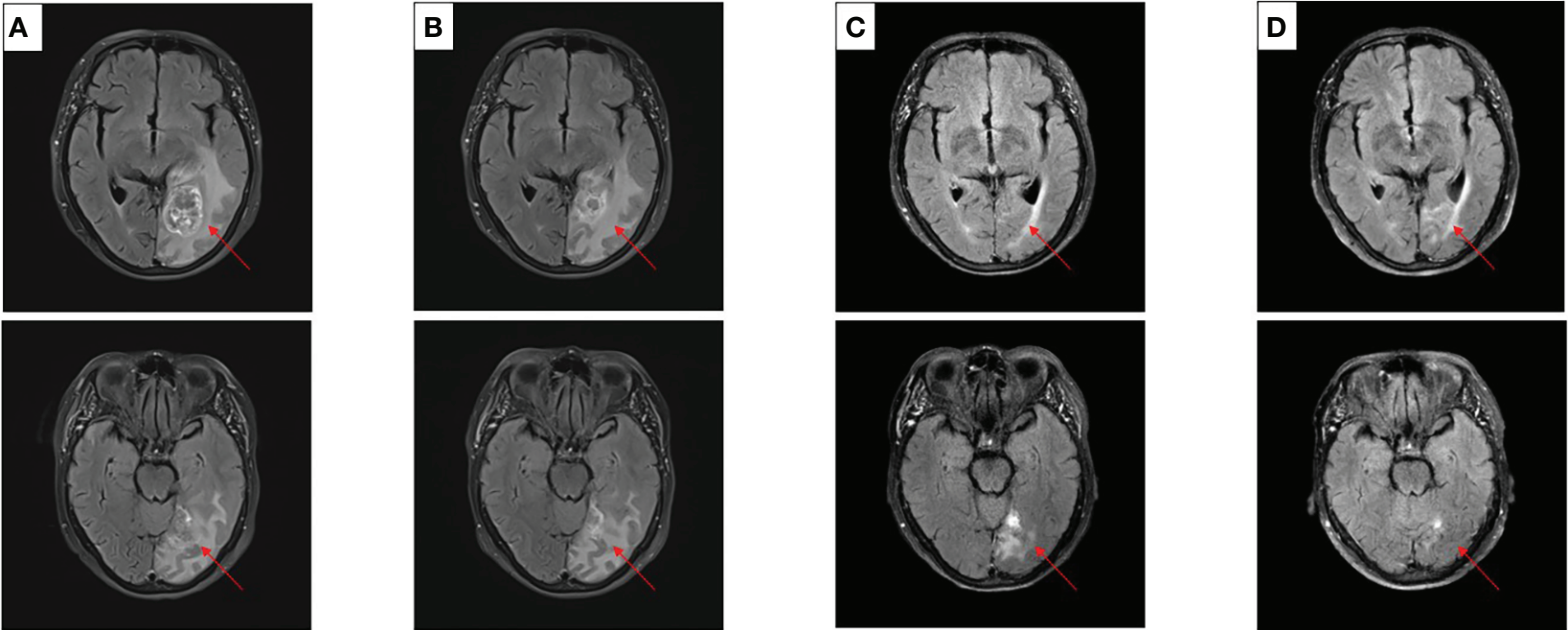
Craniocerebral MRI about clinical response to dacomitinib therapy at different times. **(A)** Baseline before dacomitinib treatment. **(B)** Brain metastases partial response (PR) after 2 months. **(C)** Brain metastases PR after 4 months. **(D)** Brain metastases PR after 6 months.

**Figure 4:**
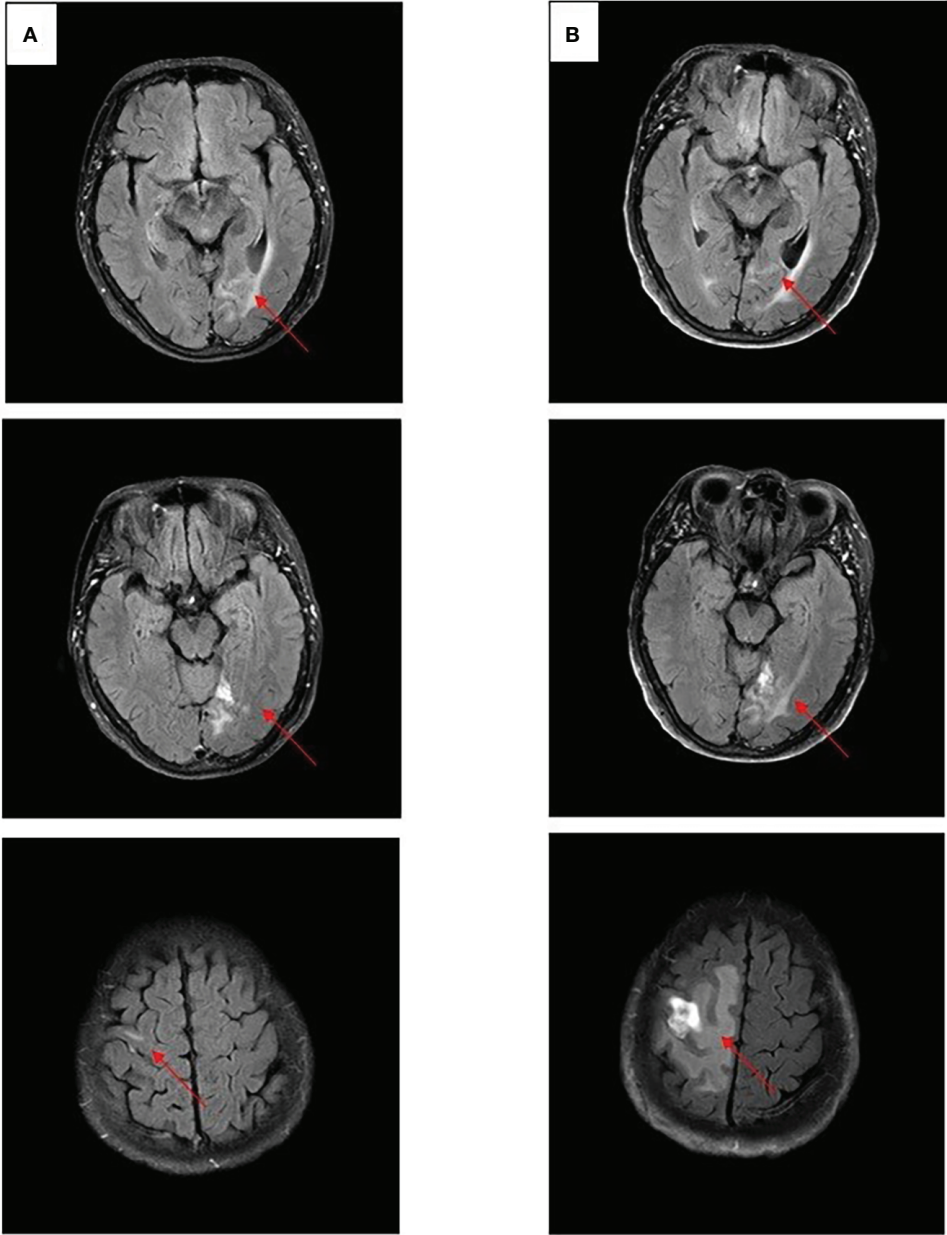
Craniocerebral MRI about clinical response to dacomitinib therapy at different times. **(A)** Brain metastases still PR after 12 months. **(B)** Brain metastases PD after 17 months.

**Figure 5:**
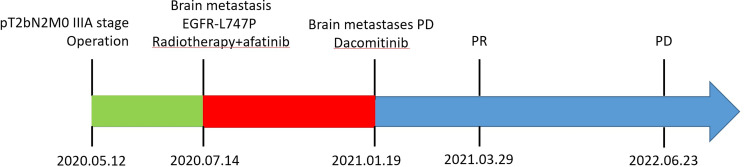
Treatment history and genomic results during the patient’s clinical course.

After experiencing dizziness and headache, the patient was reexamined by brain MRI on 19 January 2021, which revealed a new brain metastasis in the left occipital lobe (**Figure 3A**). The patient refused radiotherapy, chemotherapy, and immunotherapy, and started oral dacomitinib treatment with a dose of 30 mg daily. After several days, the patient’s neurological symptoms were significantly relieved. The patient underwent MRI 2 months later, and found that the lesion was significantly reduced, the efficacy achieved partial response (PR) (**Figure 3B**), and follow-up MRI at 4 and 6 months showed that the lesion continued to shrink and almost disappeared (**Figures 3C, D**). The patient continued to receive dacomitinib until new right parietal lobe brain metastases were identified on 23 June 2022 (the cutoff day, **Figures 4A, B**), and the progression-free survival (PFS) was 17 months (**Figure 5**). No obvious adverse reactions have been observed during the patient’s medication.

## Discussion

The L747P mutation in exon 19 of EGFR is rarely reported. One reason may be that the PCR kits are commonly used in clinical practice in most hospitals, and the results are used to guide doctors in treating patients. However, by using these methods, the L747P mutation may be incorrectly identified as 19del or false-negative as wild type, resulting in incorrect information for the guidance of clinical management (5, 6). Compared with the PCR detection method, NGS detection can more accurately identify the patient’s EGFR mutation, reduce the misidentification of rare mutations, and provide more information to guide the clinical treatment strategies.

Most EGFR mutations are highly sensitive to EGFR-TKI, but some rare EGFR mutations, such as the L747P mutation, reduce sensitivity to some EGFR-TKI due to their special conformation. Yoshizawa’s study (7) use binding free energy calculations and microsecond-timescale molecular dynamics (MD) simulations, revealing that the L747P mutation considerably stabilizes the active conformation through a salt-bridge formation between K745 and E762, and markedly decreased the van der Waals interaction between EGFR tyrosine kinase and gefitinib, resulting in resistance.

In the previous case report, patients with the uncommon EGFR mutations L747P were resistant to both first-generation (1G) and third-generation (3G) EGFR-TKI (7–11). Second-generation (2G) TKI afatinib shows better antitumor activity and achieved numerically longer PFS than that with 1G or 3G TKIs (3, 4, 11). In the binding affinity to EGFR TKIs by kinetic simulations, 1G TKIs (gefitinib, erlotinib, and icotinib) showed the worst binding affinity to the p.L747P mutation, and 3G TKI osimertinib showed moderate binding affinity. In contrast, the 2G TKIs (afatinib and dacomitinib) conferred the best binding affinity. In the subsequent cellular kinase inhibition assay and mice xenograft experiment, dacomitinib also achieved similar results to afatinib. 2G TKI (afatinib and dacomitinib) showed a lower IC_50_ value of Ba/F3 cell and A431 cell expressing p.L747P compared with 1G or 3G TKIs (**Table 1**), indicating that dacomitinib is likely to be as effective against L747P as afatinib (7, 11).

**Table 1:** Kinase inhibition activity of diverse EGFR TKIs against Ba/F3 and A431 p.L747P cell lines (7, 11).

IC_50_ (nmol)	A431 p.L747P	Ba/F3 p.L747P
Gefitinib	147.3	45.3
Erlotinib	167.3	67
Afatinib	6.7	0.8
Dacomitinib	5.2	1.8
Osimertinib	80.9	12.6

Moreover, compared with afatinib, dacomitinib has potent efficacy for the brain metastases. In the case series study of 14 central nervous system (CNS) metastasis in EGFR-positive NSCLC patients, the objective response rate (ORR) was 92.9% and the disease control rate (DCR) was 100% (12).. Another study also achieved 100% DCR in eight CNS metastasis patients (13). Therefore, dacomitinib may be more effective than afatinib for CNS metastases in patients with L747P.

In few case reports (14, 15), osimertinib may have a certain efficacy against EGFR L747P mutations, but in these cases, osimertinib is only effective in first-line use, and there is no clinical evidence that osimertinib can be effective after afatinib resistance. Therefore, although osimertinib can also achieve significant exposure in the brain, the treatment for L747P mutation should still be based on 2G EGFR TKIs as far as the existing conclusions are concerned.

In the present case, the patient had an EGFR L747P mutation, and she paid more attention to the quality of life and feared antitumor therapy, so she refused to accept chemotherapy and radiotherapy for BMS and was only willing to accept oral targeted therapy. We found that she was resistant to afatinib but responded to dacomitinib and achieved PR on brain metastases for 17 months.

Fortunately, dacomitinib worked well. This is the first case report of the effective application of dacomitinib to patients with L747P mutation, and also the first case report of the use of EGFR-TKI to achieve PR efficacy in L747P mutation patients with brain metastases.

Therefore, for L747P mutation patients with brain metastases, we can consider applying the second-generation EGFR-TKI with better effects on brain metastases like dacomitinib or using local treatment such as radiotherapy. By the way, the patient still refused chemotherapy but prepared for radiotherapy, and future treatment options remain to be further discussed.

## Conclusion

In summary, this case demonstrates that a regimen of dacomitinib can achieve significant effects in patients with EGFR L747P mutation after progression on afatinib, especially in those who have brain metastases. This provides a new therapeutic strategy for these patients.

## Data availability statement

The original contributions presented in the study are included in the article/supplementary material. Further inquiries can be directed to the corresponding authors.

## Author contributions

YL, WG, and BJ are lead authors who participated in data collection, manuscript drafting, table/figure creation, and manuscript revision. CH are senior authors who aided in drafting the manuscript and manuscript revision. FY and JW are the corresponding authors who initially developed the concept and drafted and revised the manuscript. All authors contributed to the article and approved the submitted version.

## Funding

This work was supported by the National Major Scientific and Technological Special Project for “Significant New Drugs Development” (2020ZX09201005) and the Science and Technology Planning Project of Xiamen City (3502Z20214ZD1011).

## Conflict of interest

The authors declare that the research was conducted in the absence of any commercial or financial relationships that could be construed as a potential conflict of interest.

The handling editor declared a shared affiliation, though no other collaboration with the authors at the time of the review.

## Publisher’s note

All claims expressed in this article are solely those of the authors and do not necessarily represent those of their affiliated organizations, or those of the publisher, the editors and the reviewers. Any product that may be evaluated in this article, or claim that may be made by its manufacturer, is not guaranteed or endorsed by the publisher.
